# Dephasing Processes in the Molecular Dye Lumogen-F Orange Characterized by Two-Dimensional Electronic Spectroscopy

**DOI:** 10.3390/molecules27207095

**Published:** 2022-10-20

**Authors:** Mattia Russo, Kirsty E. McGhee, Tersilla Virgili, David G. Lidzey, Giulio Cerullo, Margherita Maiuri

**Affiliations:** 1 Physics Department, Politecnico di Milano, Piazza Leonardo da Vinci 32, 20133 Milan, Italy; 2 Institute for Photonics and Nanotechnologies (IFN), National Research Council-CNR, Piazza Leonardo da Vinci 32, 20133 Milan, Italy; 3 Department of Physics and Astronomy, The University of Sheffield, Hicks Building, Hounsfield Road, Sheffield S3 7RH, UK

**Keywords:** decoherence, ultrafast spectroscopy, two-dimensional electronic spectroscopy, dye molecules, spectral diffusion

## Abstract

Molecular dyes are finding more and more applications in photonics and quantum technologies, such as polaritonic optical microcavities, organic quantum batteries and single-photon emitters for quantum sensing and metrology. For all these applications, it is of crucial importance to characterize the dephasing mechanisms. In this work we use two-dimensional electronic spectroscopy (2DES) to study the temperature dependent dephasing processes in the prototypical organic dye Lumogen-F orange. We model the 2DES maps using the Bloch equations for a two-level system and obtain a dephasing time T_2_ = 53 fs at room temperature, which increases to T_2_ = 94 fs at 86 K. Furthermore, spectral diffusion processes are observed and modeled by a combination of underdamped and overdamped Brownian oscillators. Our results provide useful design parameters for advanced optoelectronic and photonic devices incorporating dye molecules.

## 1. Introduction

Organic dye molecules find a wide range of applications in optoelectronics and photonics, from active media in lasers [1], to fluorescent labels in confocal microscopy [2] and light emitters in displays [3]. Besides these technological applications, dye molecules are also of great interest for fundamental research studies. In recent years, single organic molecules have witnessed an increasing use in quantum technologies due to their favorable properties such as: small size, easy spectral tunability thanks to chemical design and synthesis, high photoluminescence quantum efficiency and high degree of coherence when cooled to low temperatures [4]. When placed inside an optical microcavity, upon strong coupling between excitonic and photonic modes, organic dyes have also been shown to form cavity polaritons [5,6], which are hybrid light—matter quasi particles. The lifetime of such hybrid states is defined both by the rate at which photons ‘leak’ from the cavity, and by the rate at which light and matter mutually dephase. Thus, the choice of molecular materials having long dephasing times may allow for enhanced polariton lifetimes, thereby facilitating the build-up of polariton populations and the emergence of non-linear phenomena. For example, the recently demonstrated polariton condensation [7] and super-extensive light absorption important for the development of Dicke quantum battery [8]. In summary, both cavity polaritonics and the development of quantum technologies, in which information is imprinted on a single molecule in the form of an excitonic qubit, critically require the characterization of the dephasing processes in dye molecules.

Organic dye molecules can be described with different models based on their complexity [9,10]; in this work, the dye under study can be approximated to the first order of as a two-level system (TLS), with the energy of the transition from the ground (|1〉) state to the excited (|2〉) state, $\hbar \omega_{21}=E_{2}-E_{1}$, falling in the visible range. Upon excitation of a TLS, a quantum coherent superposition of ground and excited states is created, described by the wave function $|\psi \rangle =c_{1}|1\rangle +c_{2}\;|2\rangle$, with ${\left|c_{1}\right|}^{2}$ and ${\left|c_{2}\right|}^{2}$ the probability of finding the system in the ground and excited state, respectively. Interactions with the environment cause the decay of this superposition (decoherence processes), projecting the wave function on either the ground or the excited state. For a collection of TLSs, such as dye molecules in solution or in a film, the quantum state is more appropriately represented by the density operator ρ, with its off-diagonal elements $\rho_{21}={\rho_{12}}^{*}$ describing collective coherences and the diagonal terms $\rho_{11}$ and $\rho_{22}$ describing populations of the states [11]. In this case, dynamic interactions with the environment cause the decay of the macroscopic polarization, which can be phenomenologically described within the Bloch equation [11]. For an ensemble of TLSs all with the same transition energy $\hbar \omega_{21}$, the absorption spectral profile is Lorentzian with linewidth inversely proportional to the dephasing time $\Delta \omega =2/T_{2}$, and the system is said to be homogeneously broadened. Moreover, the TLS can have a distribution of transition energies $\Delta \omega_{21}$, often described by a Gaussian function, which, combined with dynamic perturbations, describes a situation known as inhomogeneous broadening. In this case, the absorption spectrum is the convolution between the Lorentzian linewidths of the individual transitions and the Gaussian distribution of their energies, resulting in the so-called Voigt profile.

Several nonlinear spectroscopy techniques have been used to measure the dephasing time of molecular ensembles. In the frequency domain, spectral hole burning [12] consists of the use of an intense narrowband pump pulse to excite the molecules with transition frequencies close to the pump pulse frequency, thus saturating these transitions and burning a hole in the absorption spectrum. This is then detected by a broadband probe pulse), with spectral width corresponding to the homogeneous linewidth. The use of a narrowband pump pulse, however, unavoidably results in the loss of temporal resolution. In the time domain, non-collinear degenerate four-wave-mixing spectroscopies such as two- and three-pulse photon echo (2PPE/3PPE) [13] enable dephasing times to be determined with the highest possible temporal resolution. In photon echo spectroscopies, a first pulse creates a polarization in the sample, and a second non-collinear pulse, delayed by the coherence time t_1_, interferes with this polarization, generating a population grating which diffracts either the second pulse itself (in 2PPE) or a third pulse delayed by the so-called population or waiting time t_2_ (in 3PPE), resulting in the emission of a third-order nonlinear signal E^(3)^(t_1_, t_2_, t_3_), where t_3_ is the detection time. In 2PPE/3PPE the diffracted signal is integrated in time, and its energy is measured as a function of the delay t_1_, with the signal decay time constant providing a direct measurement of the dephasing time. Often, when the decay time of the signal is comparable to or shorter than the duration of the excitation pulses, one uses the 3PPE peak shift approach [14], which consists of measuring the temporal delay between the maxima of the 3PPE signals emitted in different phase-matched directions.

Two-dimensional electronic spectroscopy (2DES) [15,16] is an advanced version of 3PPE in which the third-order nonlinear signal emitted by the sample following excitation by the three pulses is fully measured in amplitude and phase by spectral interferometry with a fourth pulse, the local oscillator (LO), which is phase-locked to the third pulse. The nonlinear signal is usually spectrally resolved by a spectrometer that performs a Fourier transform (FT) with respect to the detection time t_3_, to obtain the detection energy axis $\hbar \omega_{3}$. By performing an additional FT of the signal with respect to the coherence time t_1_, which requires interferometric stability of this delay as well, one obtains the excitation energy axis $\hbar \omega_{1}$, with the final dataset corresponding to a 2D map correlating excitation ($\hbar \omega_{1}$) and detection ($\hbar \omega_{3}$) energies for a fixed value of the waiting time t_2_. Thanks to the FT approach, 2DES simultaneously combines very high temporal and spectral resolution. The diagonal peaks in the purely absorptive 2DES maps provide the most direct information on homogeneous and inhomogeneous broadening and how they evolve in time, i.e., on spectral diffusion processes [17]. In the case of a collection of TLSs with different transition energies (large inhomogeneous broadening limit) the 2DES peak will appear strongly elongated along the excitation axis, while for pure homogeneous broadening the peak will be symmetric. When the correlation between excitation and detection frequencies is lost, as in the case of spectral diffusion, the peak becomes progressively more symmetric.

In this paper we exploit 2DES to study the dephasing processes in the prototypical small dye molecule Lumogen-F orange (LFO), which was recently shown to undergo super-absorption when embedded in a microcavity [8]. We study LFO in solution and embedded in a polymer matrix, and in the latter case we perform a temperature dependent study. We introduce a model that simultaneously fits the homogeneous and the inhomogeneous broadening and extracts the dephasing time from the absorptive 2DES maps. Finally, we use our model to describe the spectral diffusion dynamics at different temperatures and to reconstruct the steady-state absorption spectrum of the molecule.

## 2. Results and Discussion

### 2.1. Sample Characterization

**Figure 1:**
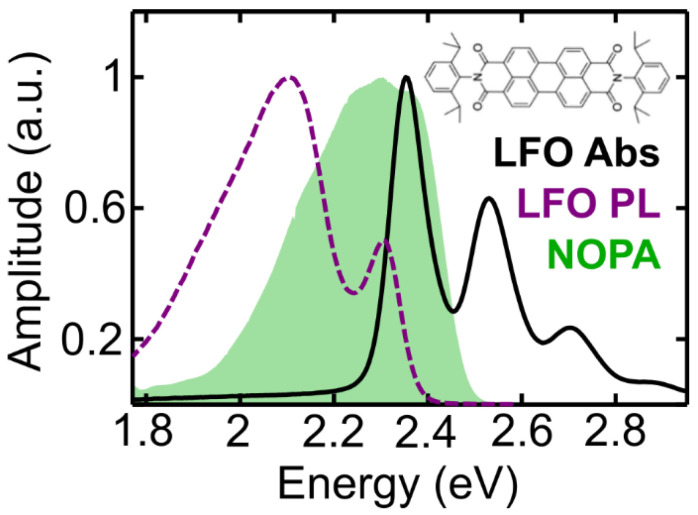
Static absorption (black solid line) and photoluminescence (violet dashed line) spectra of LFO in PS (10% concentration by mass), together with the laser pulse spectrum (shaded green line) obtained from the non-collinear optical parametric amplifier (NOPA) system. The inset shows the molecular structure of LFO.

Figure 1 shows the experimental static absorption (black line) and photoluminescence spectra (violet dashed line) of a 200 nm film of LFO (10% by mass) dispersed in a transparent Polystyrene (PS) matrix. The spectrum of the pulses (green area) completely covers the 0-0 LFO absorption peak at 2.34 eV, and the 0-0 emission peak which is positioned at lower energy. The peaks at 2.55 and 2.7 eV represent vibronic replicas of the main 0-0 transition. The broad emission band centered around 2.1 eV results from intermolecular aggregates.

To characterize homogeneous spectral broadening, one would ideally like to study isolated molecules to avoid aggregation effects, which can introduce intermolecular interaction effects. For this reason, our experiments compare LFO dispersed into either a PS or a polynorbornene (PN) matrix to form a thin film with LFO in solution. Figure S1 in the Supplementary Materials compares the absorption spectra of LFO in PN, PS and in a dichloromethane (DCM) solution. The spectra recorded for the LFO/PS film and the solution are similar in terms of peak absorption energy and linewidth, while a broader linewidth is detected in the LFO/PN film. This indicates the presence of more aggregated states and more pronounced inhomogeneous effects in the LFO/PN film. The different level of aggregation is also confirmed by the ultrafast transient absorption (TA) measurements (see Figure S2 in the Supplementary Materials), in which ground state bleaching (GSB) and stimulated emission (SE) signals decay faster for LFO/PN compared to those in the LFO/PS film and the LFO solution. These results suggest that the level of aggregation in LFO/PS film is similar to the one of LFO in DCM solution and low enough that we can consider the molecules to be isolated, allowing us to study homogeneous effects and dephasing lifetimes. The different level of aggregation is also confirmed by the TA measurements (see Figure S2 in the Supplementary Materials) in which GSB and SE signals have a prevalent bimolecular decay for the LFO/PN sample with respect to the slow monomolecular component, indicating the presence of many intermolecular interactions in this film. The opposite happens in the LFO/PS film and the LFO solution, indicating that in those two samples the molecules are prevalently isolated.

### 2.2. Two-Dimensional Electronic Spectroscopy of LFO

**Figure 2:**
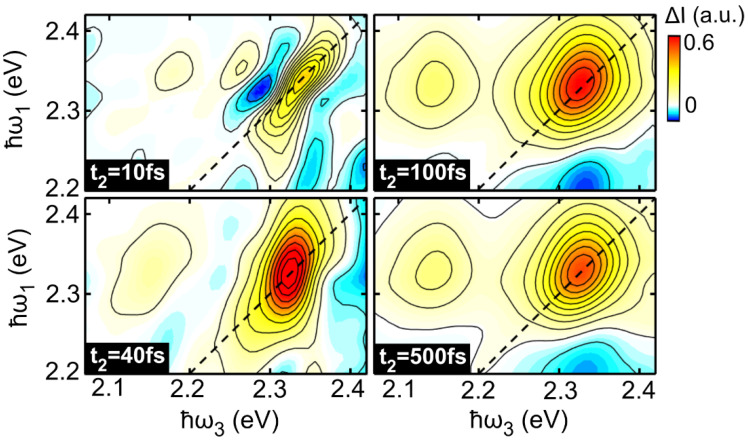
Purely absorptive 2DES maps of 10% LFO in PS at room temperature at population times t_2_ = 10 fs, 40 fs, 100 fs and 500 fs. The diagonal is shown using a dashed black line.

Figure 2 reports four 2DES maps for the LFO/PS film recorded at t_2_ = 10 fs, 40 fs, 100 fs and 500 fs at a temperature of 295 K. At t_2_ = 10 fs the 2DES map is characterized by a diagonal positive peak ($\hbar \omega_{1}$= 2.34 eV, $\hbar \omega_{3}$= 2.34 eV), corresponding to a GSB signal centered at the 0-0 absorption peak of LFO. This peak is strongly elongated along the diagonal and the mismatch between the diagonal and anti-diagonal linewidths reflects the homogenous and inhomogeneous contributions to the absorption broadening, respectively [18]. At t_2_ = 40 fs the anti-diagonal linewidth of the GSB peak increases, and a positive cross peak appears at $\hbar \omega_{3}$= 2.15 eV, reflecting the SE signal. At later times (t_2_ = 100 fs and 500 fs), the diagonal peak becomes progressively more circular. This line shape evolution is correlated with spectral diffusion in which the system loses the memory of its initially excited state. Similar spectra are obtained for LFO in solution (see Figure S3 in the Supplementary Materials).

### 2.3. Temperature Dependent Dephasing Rate

Different experimental techniques have previously been implemented to measure the dephasing time of dye molecules, including spectral hole burning, 3PPE [19] and Raman spectroscopy [20]. In 2DES measurements, the dephasing time is extracted by analyzing the widths of the 2D diagonal peak along the diagonal and anti-diagonal directions at t_2_ delays close to zero. Usually, models separately fit the two linewidths in the approximation of a large inhomogeneous broadening [21]. In such case, the homogeneous broadening only affects the anti-diagonal linewidth of the peak (inhomogeneous broadening only affects the diagonal linewidth) and it can therefore be modeled as a Lorentzian (Gaussian) function. Other models describe the absorption line shapes by including homogeneous and inhomogeneous contributions in both the diagonal and anti-diagonal line shapes of a 2D rephasing map, as in references [22,23]. The 2DES experimental configuration adopted in our work allows the detection of the purely absorptive signal (sum of rephasing and non-rephasing signals), which represents an advantage for the line shape analysis, as it avoids possible phase twist effects along the anti-diagonal direction that appear in rephasing 2DES maps [24]. Details of the model function are reported in the Methods paragraph.

**Figure 3:**
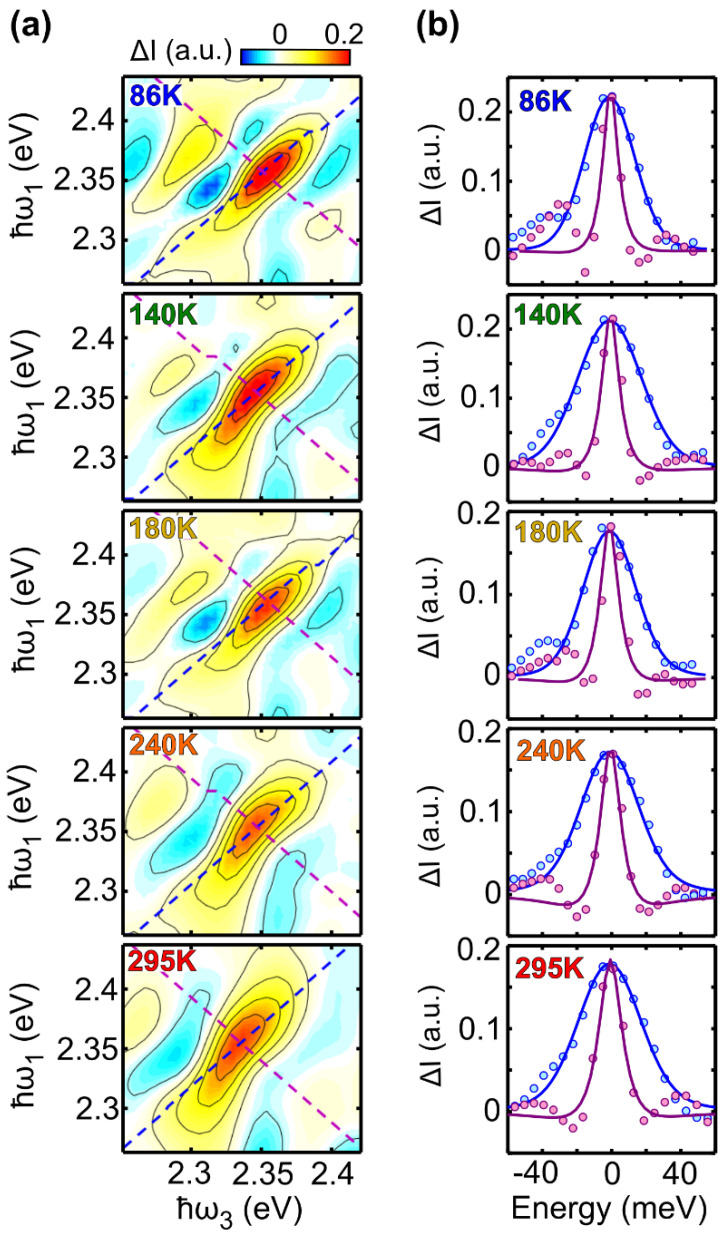
Temperature dependent linewidth analysis: (**a**) purely absorptive 2DES maps around the absorption peak at 2.34 eV at population time close to zero and at temperatures 86 K (t_2_ = 5 fs), 140 K (t_2_ = 10 fs), 180 K (t_2_ = 5 fs), 240 K (t_2_ = 5 fs) and 295 K (t_2_ = 10 fs); dashed lines represent data points that better approximate the diagonal and antidiagonal lines, not always straight due to different energy resolution along excitation and detection axes. (**b**) Best fit results (solid lines) and experimental data (colored dots) of the diagonal (blue curve) and anti-diagonal (violet curve) linewidth at each temperature and population time reported in panel (**a**).

Figure 3a shows temperature dependent 2DES maps acquired at early times (5–10 fs) at 86 K, 140 K, 180 K, 240 K and 295 K, in which the diagonal and anti-diagonal cuts have been highlighted using dashed lines. Such lines identify a new set of axes that are rotated by 45 degrees with respect to the excitation/detection axes, with its origin being in the center of the peak [25].

Figure 3b shows the experimental data corresponding to the diagonal (blue) and anti-diagonal (violet) cuts, overlapped with the best fit curves (solid lines) obtained using Equation (3) reported in Section 3 (corresponding residuals are reported in Figure S8). The values of the parameters γ and σ (expressed in meV units) used for the fits are summarized in Table 1; the obtained dephasing rate is in good agreement with the results obtained by Bardeen et al. on the dye molecule LD690 in poly(methyl-methacrylate) using 3PPE [19]. Figure S4 in the Supplementary Materials shows the result of the fit of the 2DES map at T = 295 K using the inhomogeneous limit model; the resulting dephasing rate (γ = 12.4 meV) is in good agreement with the values reported in Table 1.

**Figure 4:**
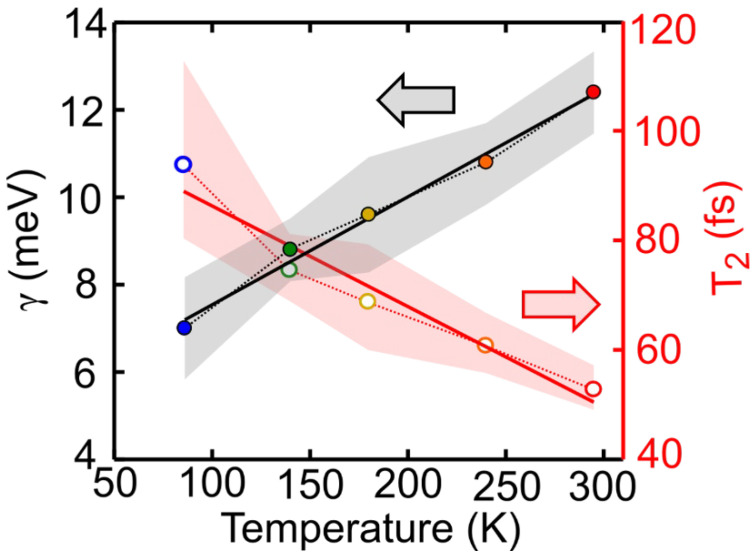
Homogeneous linewidth γ (black) and dephasing time T_2_ (red) as a function of temperature. Symbols are the estimated values of γ and T_2_ obtained from the best fit reported in Figure 3. The shaded areas reflect the error bar for each value of γ (dashed black line) and T_2_ (dashed red line) which correspond to the standard deviation value obtained from the best fit. The black and red solid lines represent the linear fit to the data.

The temperature dependence of the dephasing rate can be generally expressed using a polynomial function. When the temperature is lower than 20 K, the dephasing rate is expected to have a T^4^ dependence, while for T > 20 K, it is expected to increase linearly with temperature [19,20,26,27]. Figure 4 shows the dephasing rate as a function of the temperature, with shaded areas reflecting the sensitivity of the fit. As expected, the dephasing rate increases linearly with temperature. The best fit (black line) has a slope of 0.0247 meV/K. Figure 4 also reports the same analysis performed on the extracted dephasing time (T_2_) values, calculated using T_2_ = ħ/γ, as a function of temperature. In this case, the best fit (red line) has a slope of −0.1841 fs/K.

**Figure 5:**
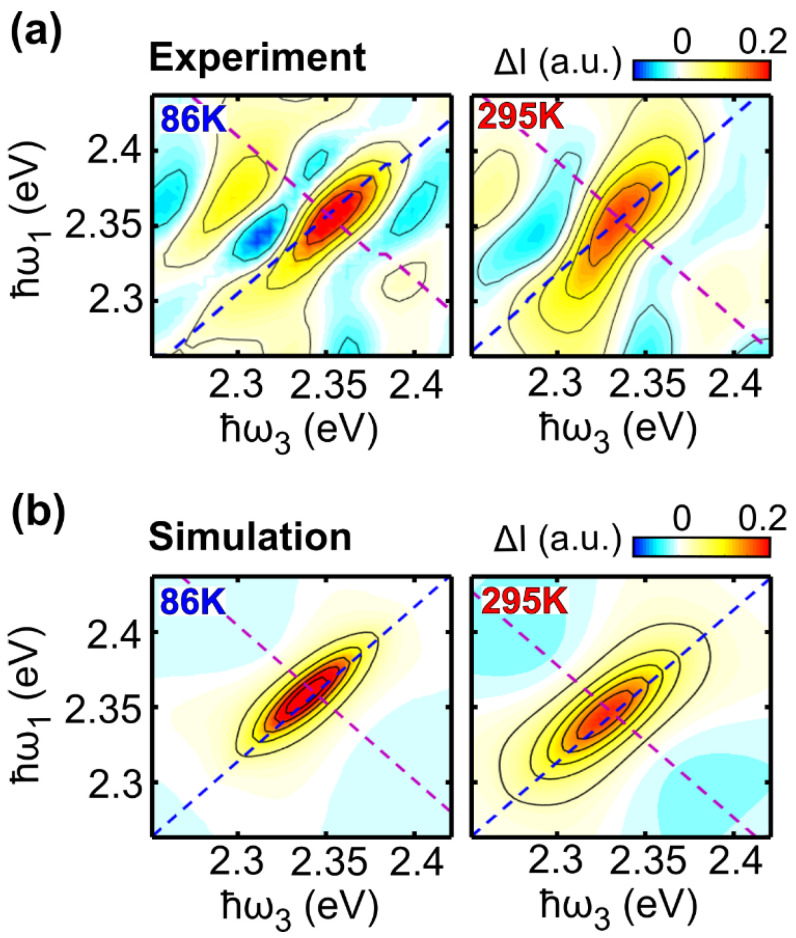
Experimental (**a**) and simulated (**b**) purely absorptive 2DES maps of LFO at 86 K (t_2_ = 5 fs) and 295 K (t_2_ = 10 fs).

Interestingly, while γ has a strong temperature dependence, we found that σ is nearly temperature independent, indicating that the temperature does not strongly affect inhomogeneous broadening. This evidence suggests that the inhomogeneous broadening arises from static contributions. Furthermore, in our fit, the amplitude of the rephasing (S_0R_) component is larger than that of the non-rephasing (S_0NR_) component at all the temperatures. This results in the elongation of the peak along the diagonal direction, with the amplitude mismatch between the rephasing and non-rephasing parts generating a negative signal along the anti-diagonal direction. These effects are clearly visible in the experimental data (Figure 3a) and are also reproduced when the 2DES maps are calculated using Equation (3), in which the parameters are substituted with the values reported in Table 1. Figure 5 compares the experimental 2DES maps at 86 K and 295 K with the calculated ones. Note that the amplitude sign change is also reproduced in the model, showing an overall good agreement and that our method allows only to reconstruct the signal associated with the diagonal peak. Several lower off-diagonal peaks have intensities comparable with the experimental noise level and are therefore not reproduced by the model.

We recall that the Equation (3) works just for a two-level system at t_2_ = 0. We performed the same analysis for LFO diluted in DCM at room temperature (results reported in Figure S5) and found that it is similar to the LFO/PS thin film.

### 2.4. Spectral Diffusion

The 2DES peak line shape displays a significant evolution with the waiting time t_2_. Indeed, at longer t_2_ times (Figure 2) the homogeneous and inhomogeneous broadening contributions are less distinguishable, due to both intermolecular and thermal-bath interactions. This causes an increase in the linewidth along the anti-diagonal direction of the 2DES maps, and consequently a change of the line shape from elliptical to circular.

To resolve the spectral diffusion, we define the Central Line Slope (CLS) [28,29,30] parameter, usually called β, an experimental value used to track the temporal evolution of the peak line shape. The CLS can be retrieved by performing a linear fit of the experimental data that identifies the maximum amplitude for every detection energy. This value of β corresponds to the angular coefficient of a linear function, and it ideally decreases from 1 (diagonal line) to 0 (horizontal line). The CLS can be shown to be proportional to the frequency fluctuation correlation function (FFCF) [11,21,28] C(t_2_), which characterizes the amplitude and time scale of spectral interaction of a molecule with its surroundings. FFCF is defined as:

(1) $$C\left(t_{2}\right)=\langle \delta \omega \left(t_{2}\right)\delta \omega (0)\rangle$$

with $\delta \omega \left(t_{2}\right)=\omega \left(t_{2}\right)-\langle \omega \rangle$.

**Figure 6:**
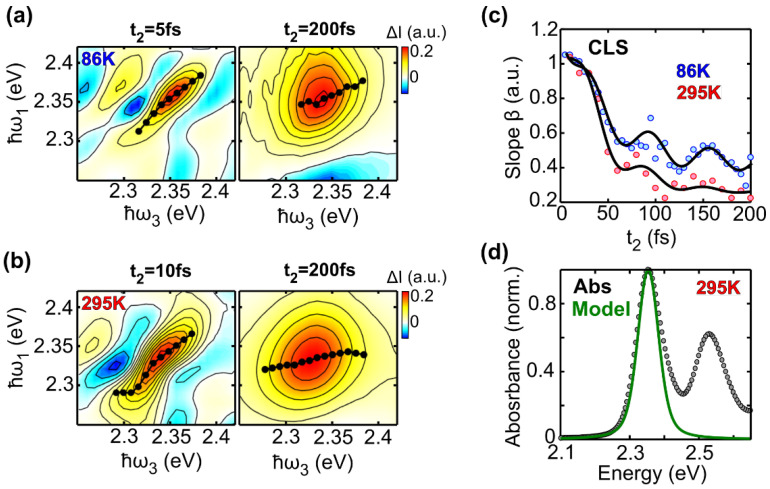
(**a**) Normalized purely absorptive 2DES maps around the absorption peak at 2.34 eV of LFO at t_2_ = 5 fs and 200 fs for T = 86 K; the black dots are obtained using the CLS algorithm; (**b**) same as (**a**), for T = 295 K; (**c**) experimental CLS dynamics (symbols) overlapped with the best fit (continuum black lines) at 86 K (blue) and 295 K (red); (**d**) Experimental (black dots) and reconstructed (green line) absorption spectrum of the LFO transition peaked at 2.34 eV.

Figure 6a compares 2DES maps at t_2_ = 5 fs and 200 fs measured at 86 K. Data extracted from the CLS algorithm are shown using black dots. At t_2_ = 5 fs, the linear fit indicates an angle close to 45° (β = 1) with respect to the detection energy axis, while at t_2_ = 200 fs the slope decreases to half of its initial value. This suggests that at T= 86 K, the timescale of spectral diffusion is longer than 200 fs, since the anti-diagonal linewidth is still narrower than the diagonal linewidth at that time. Figure 6b shows 2DES maps at t_2_ = 10 fs and 200 fs measured at room temperature. At early times, the CLS slope is similar to that measured at 86 K, however, the peak of the electronic transition has an increased diagonal linewidth. This is due to the slightly larger inhomogeneous broadening caused by increased temperature (see estimated σ values reported in Table 1). At t_2_ = 200 fs, the fit provides a line that is almost horizontal, confirming an ultrafast spectral memory loss of the system.

The spectral diffusion phenomenon is usually modeled with the FFCF C(t_2_) that represents the temporal evolution of the frequency distribution [11,21]. Due to the proportionality between β(t_2_) and C(t_2_), we can search for the best model to fit the experimental data by finding a good expression for C(t_2_). Usually C(t_2_) is expressed by a series of exponential decays in which the amplitudes and the time decays are connected to the frequency fluctuation amplitude and the correlation time [11,30,31,32]. This model is usually adopted to study slope dynamics over picosecond timescales which are far from the temporal range considered in this analysis. The dynamics over ultrafast timescales are usually characterized by an ultrafast decay over a timescale comparable with the pulse duration and possibly with molecular vibrational periods. From the analysis of the oscillatory component of the TA data, reported in Figure S6, LFO molecules have a strong vibrational mode, peaked at 540 cm^−1^, coupled to the electronic transition, that could affect the dynamics of the temporal evolution of the slope.

To model the FFCF we follow an approach implemented by Fecko et al. [33], that includes intermolecular vibrational modes described by a linear combination of underdamped and overdamped Brownian oscillators. The expression adopted in our data analysis includes: (i) an exponential decay with time constant τ_p_ comparable with the temporal resolution of the experiment; (ii) an oscillatory component describing the contribution of the vibrational mode; (iii) an offset term for the slow decay components longer than our temporal window. The final C(t_2_) function is written as follows:

(2) $$C\left(t_{2}\right)=ae^{-\frac{t_{2}}{\tau_{p}}}+be^{-\frac{t_{2}}{\tau }}\left(\cos\omega_{c}t_{2}+\left(\frac{1}{\tau \omega_{c}}\right)\sin\omega_{c}t_{2}\right)+c$$

where ω_c_ and τ are the frequency of the vibrational mode and its damping time, respectively, while the parameters a, b and c are the amplitudes related to the fast component, the vibrational mode and the long-lived exponential decay components, respectively. The last term c also includes inhomogeneous effects and hot ground state relaxation [33,34]. We fit the data by using Equation (2) as a model function with the frequency of the vibrational mode set to 540 cm^−1^. Figure 6c shows experimental values of β for LFO at 86 K (blue dots) and 295 K (red dots) compared to our best fit (solid black line) obtained with the parameters reported in Table 2 (corresponding residuals are reported in Figure S9). The results confirm a good agreement between the model and the experimental data. The dynamics of the slope parameter β at 295 K are consistent with those obtained for LFO diluted in DCM, with such a comparison reported in Figure S7.

Finally, we exploit our model to reconstruct the static absorption spectrum of LFO corresponding to the 0-0 transition at room temperature. This is done using a Voigt profile function, where we used γ and σ as parameters for the Lorentzian (homogeneous) and Gaussian (inhomogeneous) linewidth, respectively. The calculated absorption spectrum of the first peak of the vibronic progression, centered at 2.34 eV, shows a good agreement with the experimental spectrum, as shown in Figure 6d.

## 3. Materials and Methods

### 3.1. Sample Preparation

LFO (Kremer Pigmente, Aichstetten, Germany) was embedded in a PS thin-film matrix at 10% by mass. Thin films were created by first dissolving LFO in a 25 mg/mL solution of PS (Sigma-Aldrich, St. Louis, Missouri, USA)) (average molecular weight ~192,000) in DCM which was then spin-coated onto a cover-glass (Agar Scientific, Stansted, UK, 2.5 cm × 2.5 cm dimension) to produce films with a thickness of 200 nm. The spinning speed was set at 5700 rpm for a 50 s time interval. LFO was also dispersed in thin PN films at 10% by mass, with such films spin-cast from a chlorobenzene solution containing PN at a concentration of 35 mg/mL. The LFO for the ultrafast experiments in solution was dissolved in spectroscopic grade DCM (Carlo Erba–Dasit Group, Milano, Italy).

### 3.2. Two-Dimensional Electronic Spectroscopy

We performed 2DES using the partially collinear pump-probe geometry [35,36], in which the first two pulses (the pump pulses) are collinear and the third, non-collinear pulse (the probe pulse), is detected by the spectrometer. In this geometry, the phase-matched nonlinear signal is emitted along the propagation direction of the probe, which therefore also serves as a phase-locked LO (self-heterodyned configuration). In the partially collinear geometry, both rephasing and non-rephasing signals are emitted along the probe direction, allowing the direct detection of purely absorptive spectra [37].

The experimental 2DES apparatus is described in detail in reference [38]. Briefly, pump and probe pulses are generated by a Non-collinear Optical Parametric Amplifier (NOPA) [39], and pumped by an amplified Ti:Sapphire laser (Coherent Libra, Santa Clara, CA, USA) that emits 100 fs laser pulses centered at 800 nm with a 1 kHz repetition rate. The NOPA produces broadband visible pulses with a spectrum spanning from 1.7 eV to 2.5 eV (green area in Figure 1), compressed down to sub-20-fs duration using chirped mirrors. A pair of phase-locked collinear pump pulses is generated by a common-path birefringent interferometer, the Translating-Wedges-Based Identical pulses encoding System (TWINS) [40], followed by a pair of chirped mirrors to recompress the pulses to nearly transform-limited duration. The coherence time t_1_ is controlled with interferometric precision by varying the insertion of the birefringent wedges in TWINS over a range from −30 to + 250 fs. The population time t_2_ is controlled by a mechanical delay stage with scan range of up to 400 ps.

The data are processed as explained briefly in the following. A calibration procedure was applied to generate the excitation axis (ℏω_1_) following techniques from [38]. Then the acquired data were apodised in the time domain by a Gaussian filter centered at time-zero and decaying to 30% of its amplitude at t_1_ = 50 fs to reduce the noise level. Finally, the data were Fourier transformed and phase-corrected in order to retrieve the 2DES map S (ℏω_1_, t_2_, ℏω_3_). Note that the width of the adopted filter does not affect the shape of the linewidth.

The experiments were performed using a pump fluence of 50 μJ/cm^2^ with parallel polarization between pump and probe pulses. The LFO thin films were measured at different temperatures, from 77 K to room temperature. Ultrafast TA measurements were also performed on the same apparatus by fixing t_1_ = 0 fs and are reported in the Supplementary Materials. We analyzed the temperature dependence of the 2DES line shapes to extract the information related to homogeneous and inhomogeneous spectral broadening and to obtain the temperature dependent dephasing time.

### 3.3. Line Shape Data Analysis

We simultaneously fit both diagonal and anti-diagonal linewidths by using the Bloch Equations for a two-level system, as reported by Mukamel [11]. By applying the rotating wave approximation, an expression for the purely absorptive 2DES signal at t_2_ = 0 fs (without including the effects of spectral diffusion), written as the sum of the rephasing and non-rephasing contributions, is given as follows:

(3) $$S\left(t_{1},t_{2}=0,t_{3}\right)=FT\left\{\begin{array}{c} S_{0R}e^{-i\omega 0\left(t_{3}-t_{1}\right)}e^{-\left(t_{3}+t_{1}\right)\gamma }e^{-\sigma^{2}{\left(t_{1}-t_{3}\right)}^{2}/2}\\ +\\ S_{0NR}e^{-i\omega 0\left(t_{3}+t_{1}\right)}e^{-\left(t_{3}+t_{1}\right)\gamma }e^{-\sigma^{2}{\left(t_{1}+t_{3}\right)}^{2}/2} \end{array}\right\}$$

Here S_0R_ and S_0NR_ are the amplitudes of the rephasing and non-rephasing signals respectively; ω_0_ corresponds to the absorption peak frequency and delays t_1_ and t_3_ are the coherence and detection time. The term FT indicates the Fourier transform operator. The parameter γ corresponds to the dephasing rate, defined as the inverse of the dephasing time (γ = 1/T_2_), and it is connected to the homogeneous spectral linewidth. The parameter σ includes information on inhomogeneous spectral broadening. We use this function as a model to fit the experimental data, where t_1_ and t_3_ are scanned from 0 fs to 250 fs and ω_0_ is fixed to the peak position along the diagonal line (2.34 eV). All other parameters (S_0R_, S_0NR_, γ, σ) are varied to reproduce the diagonal and anti-diagonal cuts of the experimental data. This analysis was performed for the 2DES ma at t_2_ = 5 fs and 10 fs to reduce possible interference effects due to the overlap between the pulses.

## 4. Conclusions

Characterizing decoherence mechanisms in molecular dyes is important for their application in photonics and quantum technologies. Conventional spectroscopic techniques use multiple approaches to determine dephasing times and spectral diffusion processes [12,14,19,20,41,42]. Conversely, 2DES is an ideal tool for retrieving such information all together; it simultaneously differentiates the contributions from homogeneous and inhomogeneous broadening mechanisms by the shape of the diagonal peaks, while following the loss of excitation memory due to spectral diffusion, thanks to its combination of high temporal and spectral resolution.

In this study we have performed 2DES of the prototypical molecular dye LFO embedded in a polymer under low aggregation conditions, which allows intermolecular interactions to be neglected. We accurately reproduced the experimental 2DES maps via a model using the Bloch equations for a two-level system. At 295 K we obtained a dephasing rate of γ = 12.4 meV, which corresponds to a dephasing time of T_2_ = 53 fs. A similar result is obtained for the molecule diluted in DCM (γ = 11.5 meV), and by applying an inhomogeneous limit model (γ = 12.7 meV). Temperature dependent 2DES measurements from 86 K to 295 K show a linear dependence of the dephasing rate on the temperature; at T = 86 K, the dephasing rate decreases to γ = 7 meV, with a dephasing time T_2_ = 94 fs, which is nearly doubled with respect to room temperature. Measurements as a function of the waiting time show a rapid loss of excitation memory on the 200 fs timescale due to spectral diffusion, with the CLS of the peaks modeled with underdamped and overdamped Brownian oscillators combined with an ultrafast decay.

Overall, the results of our 2DES experiments in LFO are in good agreement with previous studies of dephasing processes in organic dyes. We believe that our results will provide useful parameters for the design of advanced optoelectronic and photonic devices (such as polaritonic cavities and organic quantum batteries) exploiting such molecules.

## Figures and Tables

**Table 1:** Fitting parameters to the 2DES maps shown in Figure 3a according to the model described by Equation (3) (γ: dephasing rate, σ: inhomogeneous width, S_0R_, S_0NR_: 2DES rephasing and non rephasing amplitudes).

Temperature (K)	γ (meV)	σ (meV)	S_0R_ (10^−5^)	S_0NR_ (10^−5^)
86	7	18.7	1.8	1.4
140	8.8	24	2.46	1.53
180	9.6	19.2	1.87	1.14
240	10.8	20.2	2.39	0.7
295	12.4	21.8	2.92	1.43

**Table 2:** Fitting parameters to the CLS at 86 K and 295 K shown in Figure 6c according to the model described by Equation (2), where the parameter ω_c_ is kept fixed.

Temperature (K)	a (10^−3^)	τ_p_ (fs)	b (10^−3^)	τ (fs)	ω_c_ (cm^−1^)	c (10^−3^)
86	6 ± 2	35 ± 5	1.1 ± 0.3	260 ± 35	540	3 ± 1
295	5 ± 2	35 ± 5	1.1 ± 0.4	53 ± 4	540	1.1 ± 0.4

## Data Availability

The data presented in this study are available on request from the corresponding author.

## Supplementary Materials

The following supporting information can be downloaded at: https://www.mdpi.com/article/10.3390/molecules27207095/s1, Figure S1: Static absorption and photoluminescence spectra of LFO films in polystyrene and polynorbornene; Figure S2: Transient absorption results of LFO in polystyrene (a), in solution (b) and in polynorbornene (c); Figure S3: 2DES of LFO diluted in DCM; Figure S4: Lineshape analysis results of LFO in Polystyrene obtained with inhomogeneous limit approach; Figure S5: Lineshape analysis results of LFO diluted in DCM; Figure S6: Transient absorption frequency analysis results for LFO/PS; Figure S7: CLS dynamics of LFO in polystyrene and diluted in DCM; Figure S8: Lineshape best fit residuals; Figure S9: CLS best fit residuals.

## Sample Availability

Samples of the compounds are available from the authors.
