# Supplementary material for: Dephasing Processes in the Molecular Dye Lumogen-F Orange Characterized by Two-Dimensional Electronic Spectroscopy
Source: Molecules. 2022 Oct 20;27(20):7095. doi: 10.3390/molecules27207095 (PMC9607445; doi:10.3390/molecules27207095)
Supplement: Supplementary file 1 [file molecules-27-07095-s001.zip › molecules-1954272-supplementary.pdf]

1. Static absorption and photoluminescence spectra of LFO films in polystyrene and polynorbornene

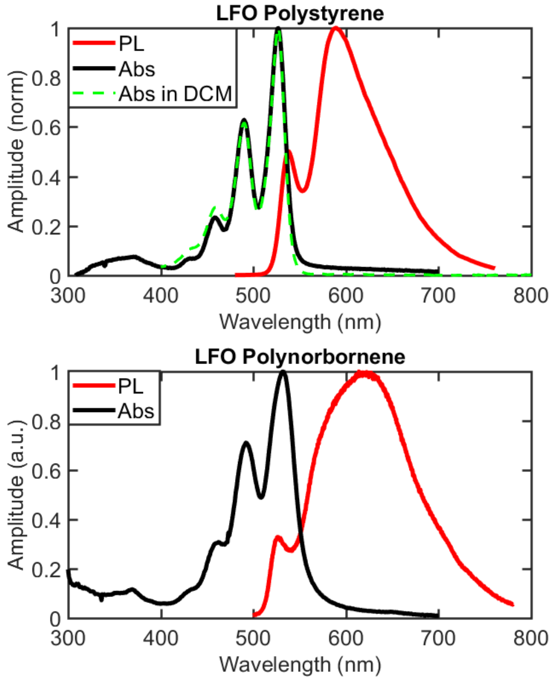

Figure S1. Static absorption (black lines) and photoluminescence (red lines) spectra of LFO films (10% concentration) in polystyrene (top) and polynorbornene (bottom). The green dashed line on the top panel represents the static absorption spectrum of the LFO diluted in DCM.

UV-vis absorption measurements were recorded using a Horiba Fluoromax 4 fluorometer equipped with a xenon lamp. For photoluminescence measurements, a 405 nm CW Thorlabs laser diode was focused onto the sample. The emitted PL was collimated and focused by a series of lenses into an optical fiber coupled to an Andor Shamrock SR-303i-A triple-grating CCD spectrometer. There was no correction done for the wavelength sensitivity. The only correction done was to measure the absorption by also measuring the absorption of a glass substrate. This was then divided by the sample spectrum. The PL spectra was not corrected in the transition dipole moment representation.

2. Transient absorption results of LFO in polystyrene, in solution and in polynorbornene

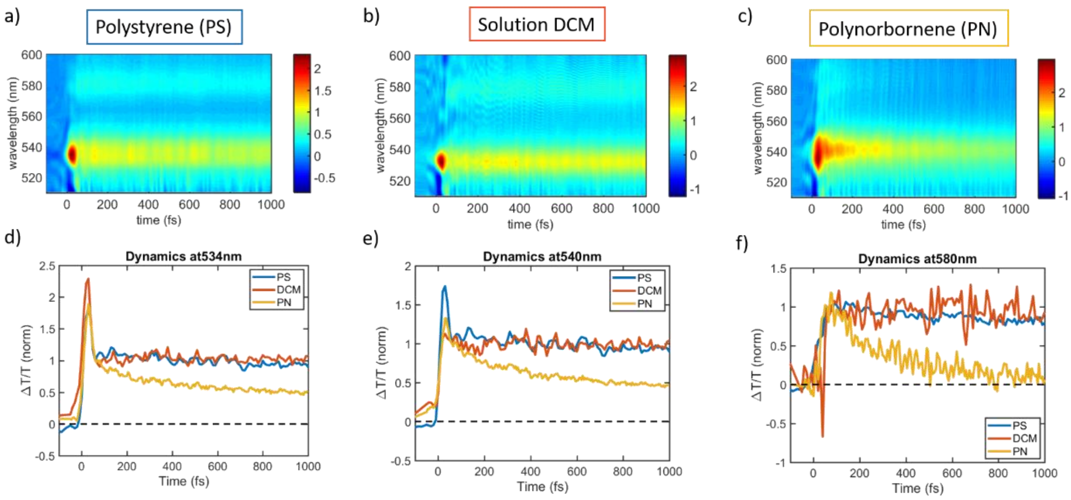

Figure S2. Transient absorption maps, as a function of wavelength and delay, of LFO in polystyrene (a), in solution (b) and in polynorbornene (c). Time traces comparison at λ=534 nm (d), λ=540 nm (e) and λ=580 nm (f).

3. 2DES of LFO diluted in DCM

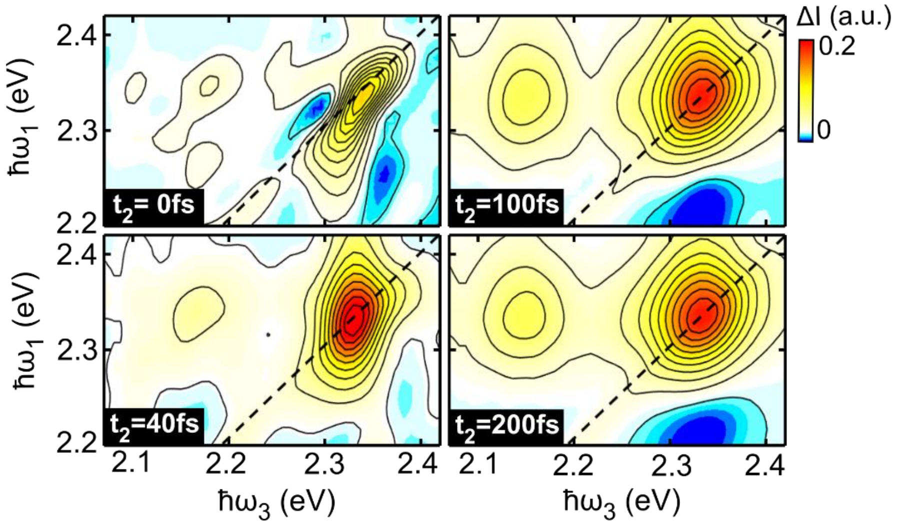

Figure S3. Purely absorptive 2DES maps of LFO 10% concentrated diluted in DCM at room temperature and at following population times: t_2=0fs, 40fs, 100fs and 200fs; the diagonal line is emphasized with a dashed black line.

4. Lineshape analysis results of LFO in Polystyrene obtained with inhomogeneous limit approach

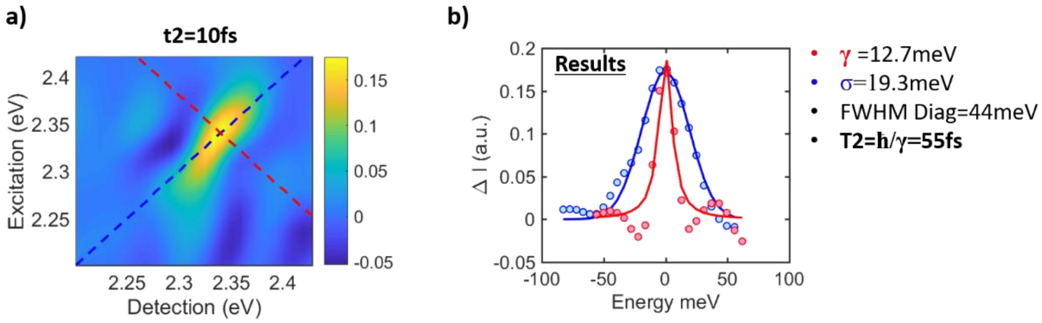

Figure S4. a) Purely absorptive zoomed 2DES map at t_2=10fs and T=295K of LFO in polystyrene in which the diagonal and anti-diagonal cuts are emphasized with blue and red dashed lines respectively; b) Diagonal (blue) and anti-diagonal (red) experimental points overlapped with the best fit obtained by using the inhomogeneous limit model. The results are reported on the right side of the plots (γ=12.7meV and σ=19.3meV).

5. Lineshape analysis results of LFO diluted in DCM

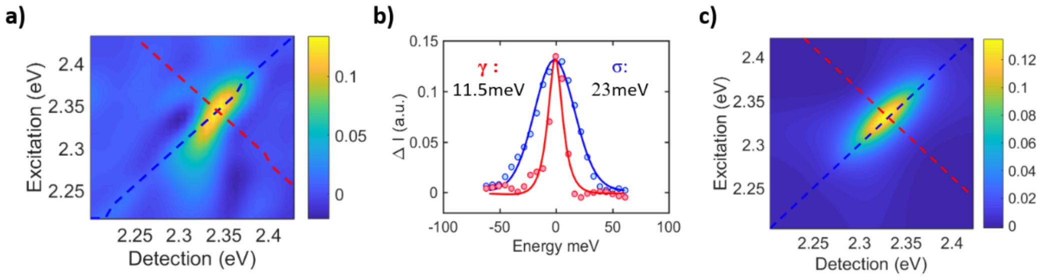

Figure S5. a) Purely absorptive zoomed 2DES map of LFO diluted in DCM (10% concentrated) at t_2=0fs and T=295K; b) Experimental (dots) and best fit (solid lines) of the diagonal (blue) and anti-diagonal (red) cuts obtained by using Eq 1 in which the final values of γ and σ are reported on the graph; c) Simulated 2DES map obtained by substituting the best fit parameters in Eq. 1.

6. Transient absorption frequency analysis results for LFO/PS

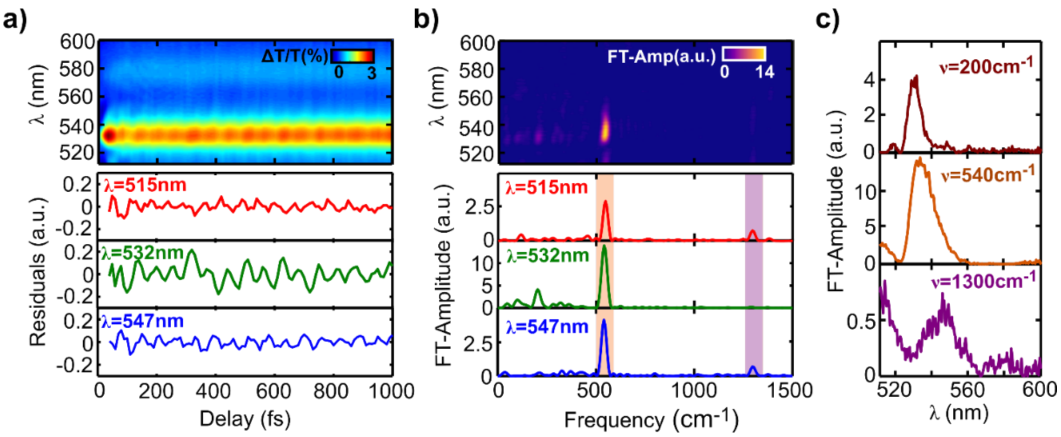

Figure S6. frequency analysis of the oscillatory component of the signal for LFO in polystyrene: panel a) transient absorption 2D map (on top) and time traces obtained by subtracting the exponential decay part at λ=515nm (red), λ=532nm (green) and λ=547nm (blue). Panel b) 2D Frequency map obtained by Fourier transforming the transient absorption data (on top) and horizontal cuts at

λ=515nm (red), λ=532nm (green) and λ=547nm (blue). Panel c) vertical cuts of the 2D frequency map in panel b) at ν=200cm^{-1} (dark red), ν=540cm^{-1} (orange) and ν=1300cm^{-1} (violet).

7. CLS dynamics of LFO in polystyrene and diluted in DCM

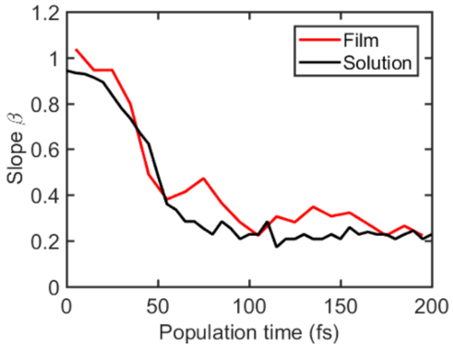

Figure S7. Comparison of CLS dynamics for LFO in polystyrene (red) and LFO diluted in DCM (black) at room temperature.

8. Lineshape best fit residuals

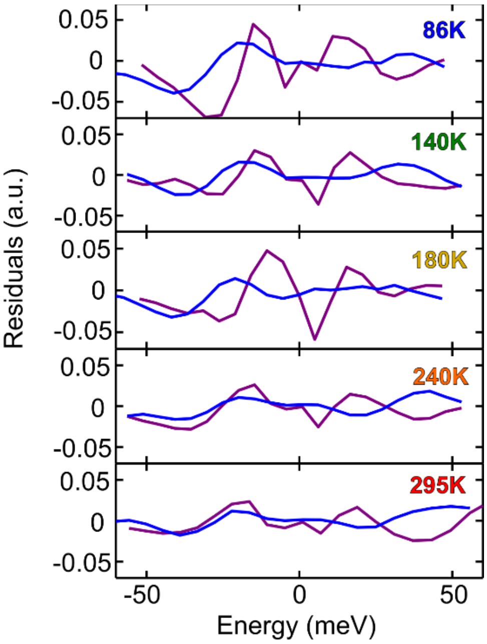

Figure S8. Diagonal (blue) and anti-diagonal (violet) best fit residuals obtained by using Eq. 1 at different temperature; starting from the top we have 86K, 140K, 180K, 240K and 295K.

9. CLS best fit residuals

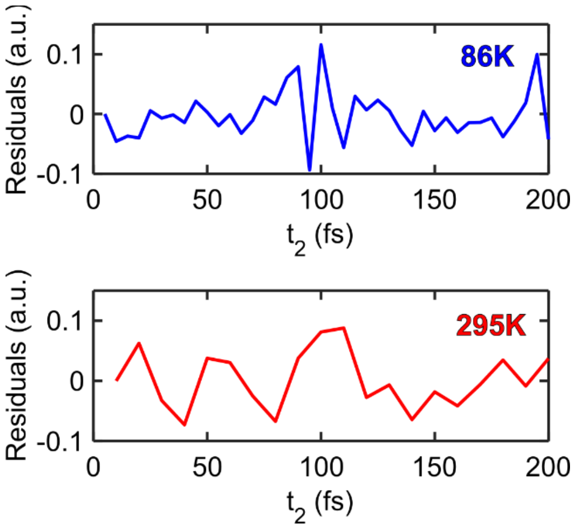

Figure S9. CLS best fit residuals obtained at 86K (top, blue) and 295K (red, bottom) by using Eq. 3
